# Calyculin A from *Discodermia Calyx* Is a Dual ActionToxin that Blocks Calcium Influx and Inhibits Protein Ser/Thr Phosphatases

**DOI:** 10.3390/toxins4100940

**Published:** 2012-10-22

**Authors:** Maja Holy, David L. Brautigan

**Affiliations:** 1 Center for Cell Signaling, University of Virginia School of Medicine, Charlottesville, VA 22908, USA; Email: mh5yu@virginia.edu; 2 Department of Microbiology, Immunology, and Cancer Biology, University of Virginia School of Medicine, Charlottesville, VA 22910, USA

**Keywords:** phosphatases PP1, PP2A, cyclin D1, calcium channels, cell cycle, MDA-MB-231, MDA-MB-468, MCF-7, ARPE-19

## Abstract

Calyculin A (Caly A) is cell permeable toxin widely used in cell biology research as an inhibitor of type 1 and type 2A protein Ser/Thr phosphatases of the PPP family. Here we tested effects of low concentrations of Caly A on proliferation of human cancer and non-cancer cell lines. We found that long-term 0.3 nM Caly A prevented G1 to S phase cell cycle progression in human Hs-68 fibroblasts and ARPE19 epithelial cells, but not human breast cancer MDA-MB-468, MDA-MB-231 and MCF7 cells. These conditions produced no change in cyclin D1 levels or in the phosphorylation of endogenous proteins. However, acute application of 0.3 nM Caly A blocked serum-induced increase in intracellular calcium levels in Hs-68 fibroblasts, but not in MDA-MB-468 breast cancer cells. We propose that subnanomolar Caly A prevents cell cycle progression because it blocks calcium uptake by fibroblasts. This probably involves non-selective cation channels and cancer cell proliferation was not affected because calcium enters these cells by other channels. Our results suggest that calyculin A has dual actions and acts as a channel blocker, in addition to its well-established effects as a phosphatase inhibitor.

## 1. Introduction

Calyculin A (Caly A) was initially isolated from a marine sponge Discodermia Calyx, collected in the Gulf of Sagami in Japan. Caly A is potent inhibitor of starfish development and acts as a cytotoxic compound against L1210 leukemia cells [1,2]. When Caly A was applied to smooth muscles it caused contractions, a response that was attributed to both an increase in intracellular calcium ion concentration as well as calcium-independent increase in phosphorylation of myosin light chain [3,4]. These observations prompted analysis of Caly A as an inhibitor of type 1 and type 2A protein Ser/Thr phosphatases of PPP family [5]. Calyculin A is one of the four types of okadaic acid class of compounds, the others being okadaic acid, microcystin-LR and tautomycin. These are potent inhibitors of the PPP family of phosphatases but do not inhibit PTP or PPM family of phosphatases [6,7]. Caly A inhibits the PPP phosphatases at low nanomolar doses and this property has been thought to account for its biological actions.

Caly A is a complex natural product with 15 chiral centers. Study of the structure-activity relationship identified several functional groups, such as the phosphate group, the hydroxyl at C-13, and the hydrophobic polyketide tail, as essential for the inhibitory action on the PPP phosphatases [1,8]. X-ray diffraction showed that Caly A folds up using H-bonds to enwrap the phosphoryl group, which is extremely resistant to chemical and enzymatic hydrolysis. This conformation of Caly A shielding the phosphate group probably accounts for membrane permeability and resistance to hydrolysis. Caly A bound to PP1 shows a quite different conformation, peeled open by interactions with side chains of the phosphatase, thereby exposing the phosphoryl group to the enzyme active site, where it binds but is not hydrolyzed [9]. This conformational change is unique among the okadaic acid class of phosphatase inhibitors and might account for the effectiveness of Caly A as an inhibitor of phosphatases in intact cells [10,11].

We have previously demonstrated that low nanomolar doses (10–50 nM) of Caly A induced T286 phosphorylation and proteasome-mediated degradation of cyclin D1 in MDA-MB-468 and MDA-MB-231 human breast cancer cells [12]. This ablation of cyclin D1 occurred within minutes at relatively low Caly A concentrations. Under these conditions there was not an increase in phosphorylation of several endogenous proteins that functioned as reporters of phosphatase inhibition. We suggested Caly A was especially effective at inhibition of a cyclin D1 T286 phosphatase, but the identity of this phosphatase remains unknown. Ablation of cyclin D1 was expected to prevent G1 to S phase cell cycle progression [13,14]. However, using flow cytometry we observed increases in either G1 or S phase populations with different breast cancer cell lines. Analysis of the effects of Caly A on cell cycle progression was hampered by the detachment and reduced survival of the cells.

To further investigate the specificity of Caly A in this study we used lower doses and compared the response of breast cancer with human Hs-68 fibroblast and ARPE-19 retinal epithelial cell lines. These cell lines were selected as commercially available (ATCC, CRL-1635 and CRL-2302) human cells that have normal karyotype and serum-dependent growth, to serve as immortalized but non-transformed controls. To our surprise we observed that at extremely low doses of Caly A (<1 nM) non-cancer fibroblast and epithelial cells were arrested and did not enter S phase, without reduction of cyclin D1 levels. In contrast, breast cancer cells were resistant and not arrested by the same doses of Caly A. This led us to investigate the basis for the effects of Caly A and we found a differential effect on serum-stimulated calcium channels in the Hs-68 fibroblasts *versus* MDA-MB-468 breast cancer cells. Caly A is often used to demonstrate the involvement of PP1 and/or PP2A in the regulation of biological processes, based on the assumption that Caly A is a selective agent for inhibition of PPP phosphatases. However, our new results show that Caly A has at least dual pharmacological effects when applied to cells at nanomolar doses and affects both calcium levels as well as phosphatase activity. 

## 2. Results and Discussion

### 2.1. Effects of Low Dose Caly A on Cell Proliferation

**Figure 1:**
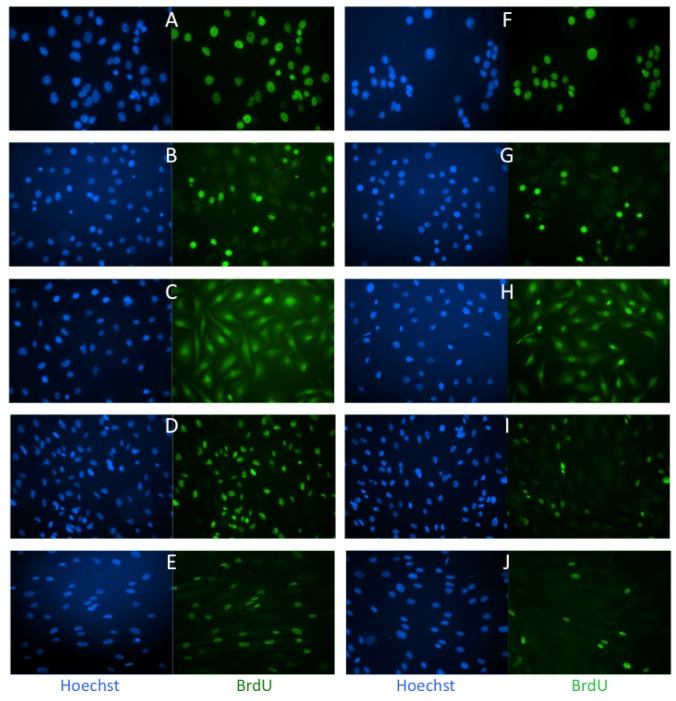
Effects of Caly A on BrdU incorporation in human breast cancer and non-cancer cells. (**A**, **F**) MCF7; (**B**, **G**) MDA-MB-468; (**C**, **H**) MDA-MB-231; (**D**, **I**) ARPE-19; (**E**, **J**) Hs-68. Cell lines were incubated with (**F**–**J**) or without (**A**–**E**) 0.3 nM Caly A for 45 h. BrdU was added 24 h before end of experiment. Cells were fixed and stained using DNA dye Hoechst and antibody against BrdU.

To analyze effects on cell proliferation we applied extremely low doses of Caly A (0.3 and 1.0 nM) that did not cause loss of cell adhesion or cell shape changes. To monitor proliferation we incubated cells with the thymidine analog bromodeoxyuridine (BrdU) and analyzed its incorporation into DNA by immunofluorescent staining. The concentration of BrdU and the duration of labeling were selected to maximize the fraction of BrdU positive cells [15]. Staining DNA with Hoechst fluorescent dye was used to determine the total number of cells. We compared three human breast cancer cell lines, MCF7, MDA-MB-231 and MDA-MB-468 without or with 0.3 nM Caly A for 45 h (Figure 1A–C,F–H). 

**Figure 2:**
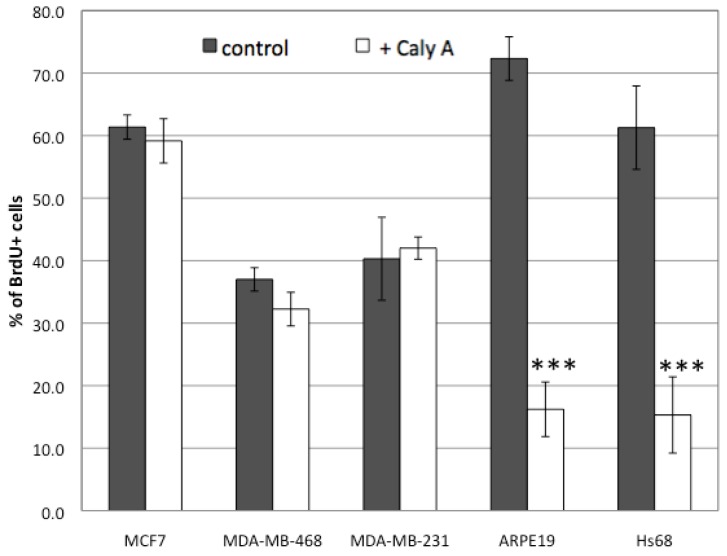
Effects of Caly A on cell cycle progression in breast cancer and non-cancer cell lines. For each cell line and condition 4 images were chosen and the number of total and BrdU positive cells were counted. The percentage of cells that incorporated BrdU was calculated for Calyculin A treated cells and controls. Plotted average value ± standard error, with *n* = 3. *** indicates *p *< 0.001 by student *t* test.

The fraction of cells that incorporated BrdU into DNA under control conditions was different among these cell lines, presumably reflecting differences in their cell cycles. All three cell lines showed no effects of this long term incubation with 0.3 nM Caly A and the results were independently replicated. This resistance to Caly A was unexpected and for a comparison we carried out the same assay using two other human cell lines, both with normal karyotype: retinal epithelial ARPE19 cells and Hs-68 fibroblasts. In contrast to the breast cancer cell lines both of these non-cancer cell lines showed a much smaller fraction of BrdU positive cells under the same conditions (Figure 1D *vs.* I, E *vs.* J). To quantify the results we plated cells in triplicate on separate coverslips and scored multiple microscopic fields on each coverslip. The BrdU-labeled cells were counted as a percentage of nuclei stained with Hoechst (set as 100%). We compared the average percentage (±SE) of BrdU positive cells and found no statistically significant difference between human breast cancer cells treated with 0.3 nM Caly A compared to controls (Figure 2). On the other hand both ARPE19 epithelial cells and Hs-68 fibroblasts displayed statistically significant (*p *< 0.001, *n* = 3) reduction in the percentage of cells that incorporated BrdU. We concluded that human fibroblasts and epithelial cells were prevented from entering the S phase of the cell cycle by low dose Caly A. In contrast the different human breast cancer cell lines were relatively resistant to Caly A. 

### 2.2. Caly A Arrests G1 to S Phase Progression without Cyclin D1 Ablation

**Figure 3:**
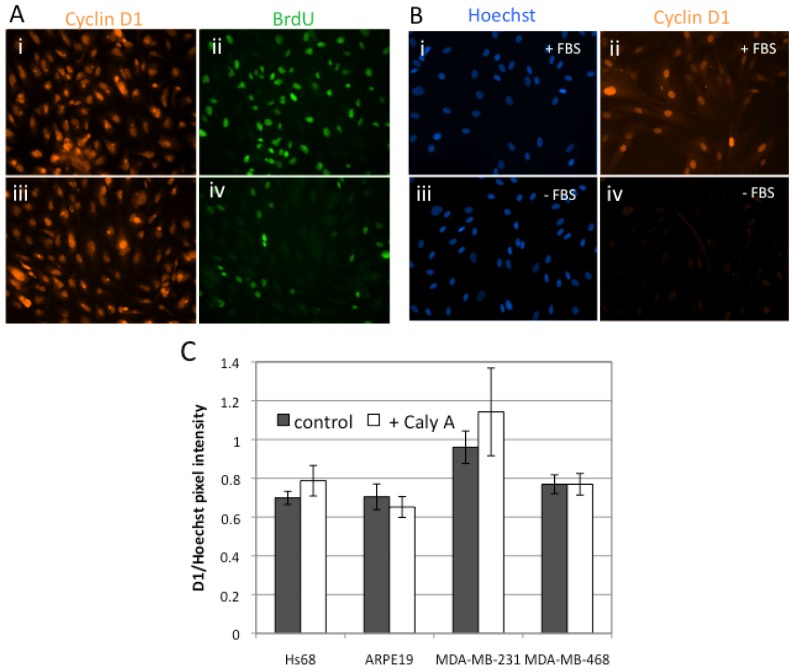
Effects of Caly A on cyclin D1 levels. (**A**) Double immunofluorescent labeling was used to analyze the levels of cyclin D1 (i, iii) and BrdU (ii, iv) in the same individual cells after 45 h incubation with (iii, iv) or without (i, ii) 0.3 nM Caly A. Representative images for ARPE19 cells are shown. Hoechst staining was shown in Figure 1; (**B**) Validation of cyclin D1 antibody specificity. Hs-68 were grown for 3 days with (i, ii) or without (iii, iv) fetal bovine serum (+/− FBS) and subsequently processed using the same staining protocols; (**C**) Graph of pixel intensity for cyclin D1 normalized to Hoechst (total DNA stain) for four different cell lines after 45 h incubation with or without 0.3 nM Caly A. Plotted average value ± standard error, with *n* = 3, not significant by student’s *t* test.

Since Caly A blocked progression into S phase, we predicted that Hs-68 fibroblast and ARPE19 epithelial cells would show reduced levels of endogenous cyclin D1. We employed double immunofluorescent labeling to assay for the levels of cyclin D1 and BrdU in the same individual cells (Figure 3A). In control experiments without Caly A most of the cells were positive for BrdU staining (Figure 3A panel ii) and every cell was positive for cyclin D1 (Figure 3A panel i). In response to 0.3 nM Caly A for 45 h the majority of cells did not stain for BrdU (Figure 3A panel iv), however all the cells were positive for cyclin D1 (Figure 3 A panel iii). There was no detectable difference in the proportion of cells positive for cyclin D1, or the levels of cyclin D1, between control and Caly A treated cells (Figure 3A panels i and iii). 

We carried out a series of control experiments to validate the specificity of cyclin D1 immunostaining. Human Hs-68 fibroblasts were incubated with or without FBS and processed using the identical staining protocol (Figure 3B). Cells cultured with FBS were positive for cyclin D1, revealing diffuse cytoplasmic staining in the all of the cells and more intense nuclear staining in some of the cells (Figure 3B panel ii). In contrast, cells cultured without FBS were detected based on Hoechst staining (Figure 3B panel iii) but showed no immunoreactivity for cyclin D1 (Figure 3B panel iv). These results show that serum was required for expression of cyclin D1 in Hs-68 fibroblasts and validated the specificity of our procedures for immunostaining for cyclin D1.

**Figure 4:**
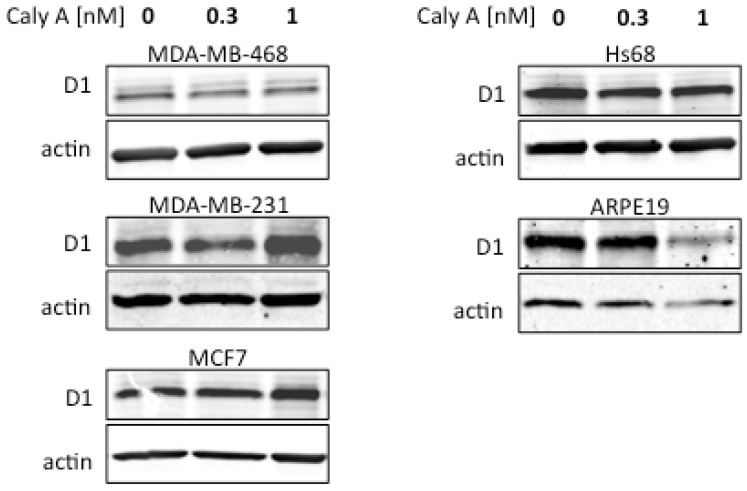
Effects of Caly A on cyclin D1 levels analyzed by Western blotting. Breast cancer and non-cancer cell lines were treated with indicated doses of Caly A for 45 h and processed for immunoblotting with cyclin D1 antibodies. Staining for actin is shown as the loading control. Data are representative of 3 independent experiments.

The effects of low dose Caly A on expression of cyclin D1 were examined in human fibroblasts, epithelial cells and two different human breast cancer cell lines, MDA-MB-231 and MDA-MB-468 (Figure 3C, *n* = 3). In each case the total pixel intensity for cyclin D1 immunostaining was normalized to DNA staining by Hoechst. This ratio was identical for three of the four cell lines and slightly higher for MDA-MB-231 cells, perhaps reflecting a higher cyclin D1 content in these cells (Figure 3C). Most important, none of these four cell lines showed any difference in this ratio between control and Caly A treated samples. Similar results were observed at both 0.3 and 1.0 nM Caly A. Our conclusion was that Caly A at these doses did not affect the expression of cyclin D1 in either cancer or non-cancer cell lines. The results were confirmed by immunoblotting for cyclin D1 (Figure 4). Cultures were incubated with 0.3 nM or 1.0 nM Calyculin A or no addition for 45 h and whole cell extracts were prepared and resolved by SDS-PAGE. Cyclin D1 antibody revealed a single predominant band of the expected size of 36 kDa. Beta-actin (43 kDa) was immunoblotted on the same filters as a loading control. Data shown are representative of 3 independent experiments.

The extended treatment of the cells with Caly A had no noticeable effect on the levels of cyclin D1 in any of the cell lines, except for ARPE19 epithelial cells that showed some reduction in D1 at the higher dose of Caly A (Figure 4). Taken together, our results show that treatment of cells with up to 1 nM of Caly A did not cause ablation of cyclin D1. As an additional control, raising the Caly A dose to 10 nM caused complete ablation of cyclin D1 within 1 h, in confirmation of our previous report [12].

### 2.3. Protein Phosphorylation in Intact Cells Treated with Caly A

**Figure 5:**
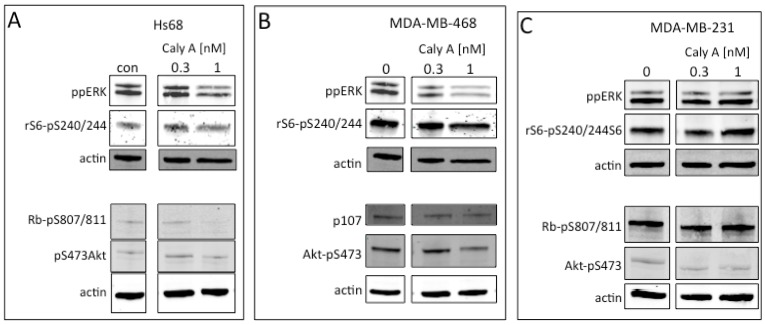
Caly A effects on protein phosphorylation. Non cancer (**A**) and breast cancer (**B**, **C**) cell lines were incubated with 0.3 or 1.0 nM Caly A for 45 h and samples were processed in parallel with untreated cells used as controls. Immunoblotting used commercial phosphosite-specific antibodies against endogenous proteins: ERK, ribosomal S6, Akt, Rb or p107. Phosphatase inhibition by Caly A would increase staining intensity. Actin was used as a loading control and images are representative of 3 separate experiments.

These results raised questions: how did 0.3 nM Caly A produce G1 arrest in the cell cycle without reduction of cyclin D1, and why no response in breast cancer cells? We tested whether the low doses of Caly A over an extended period of time would affect the phosphorylation of endogenous proteins (Figure 5). Human Hs-68 fibroblast and human breast cancer MDA-MB-468 and MDA-MB-31 cells were incubated for 45 h with 0.3 and 1.0 nM Caly A and whole cell extracts were analyzed by quantitative fluorescent immunoblotting using phosphosite specific and total protein antibodies (Figure 5). There was no change +/− Caly A in recovery of the total protein as seen by staining for actin as a loading control. Compared to untreated cells (processed in parallel as controls) the activating dual phosphorylation of ERK1/2 (ppERK), ribosomal protein S6 phosphorylation at Ser240 and Ser244, Akt phosphorylation at Ser473, and Rb or p107 phosphorylation at Ser807 and Ser811 were all unchanged (Figure 5). The different cell lines had either Rb or p107 as substrates for cyclinD1: CDK4 kinase. 

Similar results (*i.e.*, no increase in phosphorylation due to addition of Caly A to cells) were observed with the other cells lines used in this study namely, ARPE19 epithelial cells and MCF7 breast cancer cell line (not shown). If Caly A was inhibiting PPP phosphatases such as PP1 and PP2A in the cells one would have expected an increase in the phosphorylation of these endogenous proteins. The results show that at or below a concentration of 1.0 nM Caly A did not inhibit the activity of PPP protein phosphatases in living cells. 

### 2.4. Caly A Blocks Serum-Stimulated Intracellular Calcium in Hs-68 Fibroblasts

**Figure 6:**
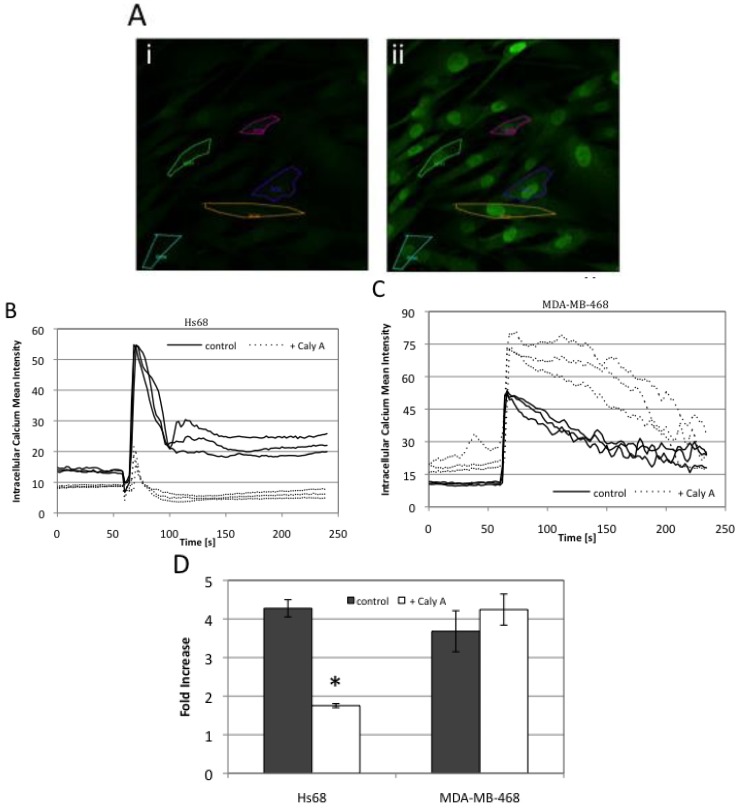
Caly A effects on serum-induced calcium influx**.** Serum-starved cells were loaded with 6 μM Fluo-3-AM for 30 min at 37 °C and intracellular calcium changes were recorded using laser scanning confocal microscopy in response to addition of serum. Images were taken every 2 s for 4 min. Specimens with Caly A (0.3 nM) were compared to controls with no addition. Hs-68 fibroblasts and MDA-MB-468 breast cancer cells were stimulated with FBS applied at 60 s after the start of recording. (**A**) An increase in fluorescence was observed in Hs-68 cells after serum stimulation. Representative images are shown, taken at 44 s (panel i) and 66 s (panel ii), which are before and after FBS addition, respectively; (**B**) Graph displaying the profile of calcium concentration over time in Hs-68 human fibroblasts. Multiple tracings of individual cells as regions of interest (ROI) for untreated controls (solid lines) and cells with 0.3 nM Caly A (dotted lines); (**C**) Graphs for multiple ROI for human breast cancer cell line MDA-MB-468, showing untreated controls (solid lines) and cells with 0.3 nM Caly A (dotted lines); (**D**) The increase in fluorescence was calculated in each cell by comparing the mean value of baseline calcium level (average between 10 s and 50 s) to the peak value. Plotted average value ± standard error, with *n* = 4 for Hs68 and *n* = 5 for MDA-MB-468. * indicates *p* < 0.05 by students *t* test.

What is targeted by subnanomolar doses of Caly A to prevent progression into S phase? This must be different in breast cancer cells compared to the non-cancer cell lines we used as controls (Hs-68 and ARPE19). We calculated that the effects were being produced by about 3 × 10^6^ molecules of Caly A per cell, a relatively low number. Some of the earliest publications describing the actions of Caly A reported effects on intracellular calcium levels [4,16,17] and calcium is known to be required for G1 to S progression [18,19,20,21]. We loaded cells with the cell permeable fluorescent calcium indicator fluo-3-AM and used laser scanning confocal microscopy to quantify intracellular calcium levels in multiple individual cells by rapid scanning of selected regions of interest (ROIs) (Figure 6A). Fibroblasts are reputed to lack voltage gated calcium channels and indeed we observed no increase in intracellular calcium in response to depolarization by potassium chloride up to 120 mM (not shown). Alternatively, other groups have shown serum stimulation of calcium levels in fibroblasts [22,23]. We observed that addition of serum to Hs-68 fibroblasts after overnight serum deprivation elicited a steep rise in intracellular calcium, followed by sustained plateau of elevated calcium (Figure 6B solid lines). This calcium entry was severely blunted and the plateau eliminated by adding 0.3 nM Caly A to the cells 1–2 min prior to addition of serum (Figure 6B dotted lines). 

When MDA-MB-468 breast cancer cells were stimulated by serum following overnight deprivation there was a sustained increase in intracellular calcium (Figure 6C solid lines, *n* = 5). The profiles of calcium concentration over time were distinctly different for fibroblasts comparing to breast cancer cells (Figure 6B *vs.* C). If 0.3 nM Caly A was added to MDA-MB-468 cells *ca.* 1 min before addition of serum there was no inhibition of the rise in intracellular calcium and shape of the curves with or without Caly A were the same (Figure 6C). These experiments were replicated 4–5 times; in each experiment typically 20–50 cells were analyzed. We calculated the relative increase in calcium concentration from the height of the serum-stimulated peak compared to the baseline levels prior to addition of FBS (Figure 6D). This analysis revealed a statistically significant (*p* = 0.05) reduction in FBS-stimulated spike in calcium levels in Hs-68 fibroblasts, compared to non-significant effect of 0.3 nM Caly A on MDA-MB-468 breast cancer cell line (Figure 6D). We concluded that the rise in intracellular calcium stimulated by serum involved different mechanisms in Hs-68 fibroblasts compared to breast cancer cells and 0.3 nM Caly A blocks the rise in calcium in fibroblasts but not in breast cancer cells.

### 2.5. Discussion

In this study we discovered that calyculin A at subnanomolar concentrations prevents G1 to S-phase progression of human Hs-68 fibroblasts and ARPE19 human epithelial cells, but not human breast cancer MDA-MB-468 or MDA-MB-231 or MCF7 cells. All the cells tolerated treatment with low dose (<1 nM) Caly A for an extended period of time (2–3 days) with no loss of viability and with no noticeable shape changes that are hallmarks of Caly A inhibition of PPP phosphatases. Our original intention was to examine effects of low dose Caly A on G1 to S phase progression and correlate that to reduced levels of cyclin D1. We previously had found that nanomolar levels of Caly A increased cyclin D1 phosphorylation at T286, triggering degradation via the proteasome [12]. But, here we observed G1 arrest without any effects on, either the levels of cyclin D1, or the phosphorylation of Rb/p107, the substrates of cyclin D1-dependent kinases. Thus, we concluded that Caly A inhibits proliferation under these conditions by a mechanism other than phosphatase inhibition. Low dose Caly A immediately blocked elevation of intracellular calcium in fibroblasts, not cancer cells, offering an inviting explanation for its differential effects on these different cell lines. We propose that the effects of the toxin on calcium levels are not dependent on its inhibition of protein phosphatases, but involve blockade of ion channels. This reveals a new mechanism of Caly A and suggests there are multiple molecular targets for the toxin. Furthermore, if Caly A is preventing calcium-dependent signaling then it implies calcium is not required for the assembly, activation or control of cyclin D1-dependent kinases. 

Early studies found Caly A at concentrations ranging from 10 nM–1 μM caused contraction in the smooth muscles of guinea pig (taenia coli or taenia caeci) and rat aorta in the presence or absence of external calcium [3,4]. It was shown that Caly A activated voltage dependent calcium channels, but this effect appeared to be independent of the Caly A induced contractions. Calcium-independent contraction of smooth muscles involves phosphorylation of myosin light chains, accomplished by inhibition of myosin phosphatase, a multisubunit form of type-1 PPP phosphatase. These early results suggested that Caly A had dual actions to produce the observed effects, but subsequent studies focused on Caly A as a protein Ser/Thr phosphatase inhibitor. Biphasic effects of Caly A also have been reported on bone resorption in neonatal mouse calvariae [24]. Caly A acted as stimulator or inhibitor, depending on time and concentration. Low concentrations (0.625, 2.5 nM) of Caly A caused stimulation of bone resorption, which was opposite to inhibition of bone resorption by higher Caly A doses (≥3.3 nM). One of the proposed explanations for these contradictory actions was that activity of the lower doses of Caly A on bone resorption could be independent of its inhibition of cellular phosphatases [24]. Our study reinforces and extends these earlier observations, and supports the idea that Caly A has dual actions on cells, affecting calcium at lower concentrations (<1 nM) and inhibiting phosphatases at higher concentrations (>1 nM).

We propose that non-selective cation channels (NSCC) could be the novel putative target for low dose Caly A. Our experiments showed no increase in intracellular calcium concentration with Hs-68 fibroblast when potassium chloride up to 120 mM was applied on cells to induce depolarization. We have attributed this to the absence or low expression of voltage-gated calcium channels in these cells. Several studies have investigated ion channel activation by serum [25,26,27] and in each case they revealed that calcium entered cells via NSCC. These channels represent a voltage-independent pathway for influx of calcium into cells. NSCC are a heterogeneous family of channels, widely expressed in nonexcitable and excitable cells and share several functional characteristics but have diverse molecular origins [28]. Stimulation of serum-deprived, quiescent Balb-c 3T3 fibroblasts with 10% fetal calf serum induced a sustained elevation of intracellular free calcium concentration. The elevation of calcium was abolished by SKF 96365, an imidazole derivative that blocks receptor-activated NSCCs. Inhibition of cell proliferation was observed when SKF 96365 was added to fibroblasts [25]. In another study with rat retinal pericytes, serum activated voltage-dependent potassium current as well as calcium influx via NSCC. In these retinal pericytes SKF 96365 diminished serum induced currents and NSCC conductance [26]. Kusaka *et al.* also studied serum-induced calcium permeability through NSCC in human and bovine Muller cells [27]. We predict that NSCC have a minimal role in serum-stimulated calcium influx in breast cancer cell line MDA-MB-468. Caly A did not affect changes in [Ca^2+^] in these cells. MDA-MB-468 cells are known to possess T-type voltage gated calcium channels [29] for calcium entry, which we propose are not affected by Caly A. Our hypothesis is that differences in expression of cation channels and not protein phosphatases account for the differential responses of cell lines to low doses of Caly A.

## 3. Experimental Section

### 3.1. Cell Culture and Reagents

MDA-MB-231 and MDA-MB-468 breast tumor cell lines were maintained in L15 media (Gibco 11415, Grand Island, NY, USA) containing 10% FBS at 37 °C in non-CO_2 _(air) conditions. All the other cell lines were cultured in a humidified 5% CO_2_ atmosphere at 37 °C. MCF7 breast cancer cells were grown in MEM (Gibco 11095, Grand Island, NY, USA) supplemented with 10% FBS, 10 μg/mL human insulin (Sigma 051M8418 St. Louis, MO, USA), 1 mM non-essential amino acids (Gibco 11140, Grand Island, NY, USA) and 1 mM sodium pyruvate (Gibco 11360, Grand Island, NY, USA). Hs-68 cells were cultured in DMEM high-glucose (Gibco 11965, Grand Island, NY, USA) with 10% FBS. All cells were passaged every 2–3 days using 1× PBS (Gibco 14190, Grand Island, NY, USA) and 0.05% trypsin-EDTA (Gibco 15400, Grand Island, NY, USA) and were grown to the maximum number of 10 passages. The following antibodies were used to perform western blotting experiments: anti-cyclin D1 (Cell Signaling Technology 2926S, Danvers, MA USA), anti-phospho44/42MAP Kinase Thr 202/Tyr204 (Cell Signaling Technology 9101S, Danvers, MA USA), anti-phosphoS6ribosomal protein Ser 240/244 (Cell Signaling Technology 2215S, Danvers, MA USA), anti-phosphoRb S807/811 (Cell Signaling Technology 9308S, Danvers, MA USA), anti-p107 (Thermo Scientific MA1-12362, Suwanee, GA, USA), anti-phospho-Akt1 Ser 473 (Upstate Cell Signaling Solutions 05-736, Lake Placid, NY, USA), anti-actin (Sigma A2103 St. Louis, MO, USA).

### 3.2. Cell Proliferation Assay and Immunofluorescence

The experiments were performed using protein coated 12 mm glass coverslips (Fisher Scientific 12-545-80, Suwanee, GA, USA), situated in wells on 24-well plate. Wells with coverslips were prepared in advance by adding 400 µL/well of fibronectin (Sigma F0895, St. Louis, MO, USA) diluted in PBS 10ug/mL, followed by incubation for 2 h at room temperature (RT) or overnight at 4 °C. Coverslips were washed 3× with PBS. Cells before plating (2.5 × 10^4^ cells/well) were mixed thoroughly in growth medium by vigorous pipetting at least 10 times in order to obtain single cell suspension. Next day Calyculin A (Sigma C7632, St. Louis, MO, USA) was diluted in medium and added to appropriate wells. To evaluate cell proliferation, 24 h before ﬁxing cells BrdU labeling reagent (Invitrogen 00-0103, Grand Island, NY, USA) was added according to provider’s instructions to final concentration of 100 μM. After that time cells were washed with PBS and ﬁxed in 3.7% formaldehyde/PBS for 15 min. After 3× PBS wash cells were permeabilized by 10 min incubation in 0.1% Triton X-100 (Fisher Scientific BP151, Suwanee, GA, USA). After 3× PBS wash, DNA denaturation was performed by coverslips incubation in 2M HCl for 20min, followed by neutralization using 0.1 M sodium borate pH 8.5 (2 min). Coverslips were blocked for 1 h with 2% BSA, 5% normal goat serum (NGS) in PBS. Mouse anti-BrdU Antibody (Ab) (Invitrogen B35128, Grand Island, NY, USA) and rabbit anti-cyclin D1 Ab (Cell Signaling Technology 2978, Danvers, MA, USA) were diluted 1:100 in 2% BSA-PBS solution and incubated overnight in 4 °C. Secondary Abs-goat anti-mouse AlexaFluor 488 (Invitrogen A11001, Grand Island, NY, USA) and donkey anti-rabbit AlexaFluor 594 (Invitrogen A21207, Grand Island, NY, USA) were diluted 1:1000 in 2% BSA/PBS and incubated for 2 h in RT. Hoechst 33342 was incubated for 15min at 1:10000. After each step fixed cells were washed 3× in 0.1% BSA/PBS.

Samples were examined and wide field images were obtained using immunofluorescence Nikon Eclipse E800 microscope equipped with a Hamamatsu 3580 camera and 20X/0.50 magnification objective (no oil immersion). OpenLab 5.5.1 Software was used for image acquisition, processing and analysis. 

### 3.3. Western Blotting

Cells were plated (depending from cell line) 1.5–3 × 10^5^ cells/well in 6-well plate. After 45 h cells were washed with PBS and whole cell lysates were prepared by applying 1× Loading Buffer (diluted from 4× stock: Tris-HCl pH 6.8, 0.25 M, SDS 8%, glycerol 30%, β-mercaptoethanol 10%, bromophenol blue 0.2%, protease inhibitor cocktail) directly to the cells after washing with PBS, followed by 10 min incubation at 100 °C. Protein samples were resolved using SDS-PAGE on precast gels from BioRad (567-1123, 567-1023 Hercules, CA, USA). Proteins were transferred from gels to nitrocellulose membranes by electrophoresis for 80 min at 25V using a semi-dry transfer apparatus (Transfer Buffer: Tris 25 mM, Glycine 192 mM, methanol 20%). Membranes were blocked with 5% bovine serum albumin (Sigma A7096, St. Louis, MO, USA) dissolved in Wash Buffer (Tris-buffered saline with 0.1% Triton X-100). Membranes were probed overnight at 4 °C with primary antibodies and washed 3× in Wash Buffer. Primary antibodies were detected using fluorescent secondary antibodies donkey anti-rabbit IR Dye 800CW (LI-COR 926-32213) and goat anti-mouse AlexaFluor 680 (Invitrogen A21058, Grand Island, NY, USA). Western blot images were acquired on an Odyssey Infrared Imaging System and software (Licor Biosciences, Lincoln, NE, USA). In each case the amount of protein loaded per well was determined to obtain the same actin signal quantified using Odyssey software. All western blot experiments were performed multiple times with similar results.

### 3.4. Live-Cell Imaging and Calcium Measurements

Cells plated onto 25 mm coverslip culture dishes (Fisher Scientific 12-545-102, Suwanee, GA, USA) situated in wells in 6-well plate (Hs-68 2 × 10^5^ cells/well; MDA-MB-468 4 × 10^5^cells/well) were grown under standard conditions. Next day media was replaced for serum-free media and cells were subjected to overnight starvation. Prior to experiments coverslips with cells were transferred to imaging chamber, loaded with 6 μM Fluo-3, AM (Invitrogen F1242, Grand Island, NY, USA)-fluorescent intracellular calcium indicator and incubated for 30 min in 37 °C. After that time the excess dye was washed off and cells were covered with DMEM high glucose phenol red free media (Gibco 21063, Grand Island, NY, USA) containing 5 mM CaCl_2_ with or without 0.3 nM Caly A. Imaging was carried out on a Leica SP5 X microscope system equipped with a 63X/1.2NA water objective. The excitation 500 nm was selected from the white light laser module and the emission signals of 510~570 nm (set through the acoustic optical beam splitter) were measured by a photomultiplier tube. Time-lapse images were taken every 2 s for 4 minutes. The treatment with serum to final concentration of 20% was typically applied at 60 s. Data acquisition and analysis were performed using the Leica LAS AF software. All experiments were performed at least 4 times, in each experiment 15–30 cells were recorded and analyzed as Regions of Interest (ROIs).

## 4. Conclusions

We propose that Caly A arrests cell proliferation because it blocks serum-stimulated calcium uptake by inhibiting non-selective cation channels. In contrast, human breast cancer cell proliferation is not blocked because calcium enters these cells by other channels. Our results suggest that Caly A is a dual action toxin that acts as a channel blocker at subnanomolar doses, in addition to its well established effects as a PPP phosphatase inhibitor at higher doses. 
